# Dynamics of cavity soliton driven by chirped optical pulses in Kerr resonators

**DOI:** 10.1007/s12200-022-00018-3

**Published:** 2022-04-27

**Authors:** Jianxing Pan, Chaoyu Xu, Zhichao Wu, Jing Zhang, Tianye Huang, Perry Ping Shum

**Affiliations:** 1 School of Mechanical Engineering and Electronic Information, China University of Geosciences (Wuhan), Wuhan, 430074 China; 2 Department of Electrical and Electronic Engineering, Southern University of Science and Technology, Shenzhen, 518055 China

**Keywords:** Cavity soliton (CS), Chirped pulse driving, Deterministic single soliton

## Abstract

Recent researches have demonstrated that pulsed driving is an effective method to increase the temporal overlap between cavity soliton (CS) and pump field, thereby increasing the pump-to-comb conversion efficiency. The amplitude-modulated inhomogeneity of the background wave causes the solitons to drift toward edges of the driving pulse. To eliminate the multiple temporal trapping positions, induced by the spontaneous symmetry breaking, we propose the chirped pulse driving for deterministic single soliton generation. We theoretically explain the physical mechanism of the chirp pulse driving, as the combination of amplitude and phase modulation. Our numerical simulations demonstrate the chirp is responsible for the single soliton generation. A detailed investigation for dynamics of CSs sustained by chirped pulses, shows the recovery of spontaneous symmetry breaking. In addition, the desynchronized chirped pulse driving is also considered here. Considering a weak chirp parameter, the desynchronization-dependent trapping position diagram is divided into multiple areas including two CSs, a single CS, two oscillating CSs, and no CS. With a sufficient chirp parameter considered, the trapping position curve becomes a monotonous function of the desynchronized drift velocity, which indicates deterministic single soliton generation.

**Graphic Abstract:**

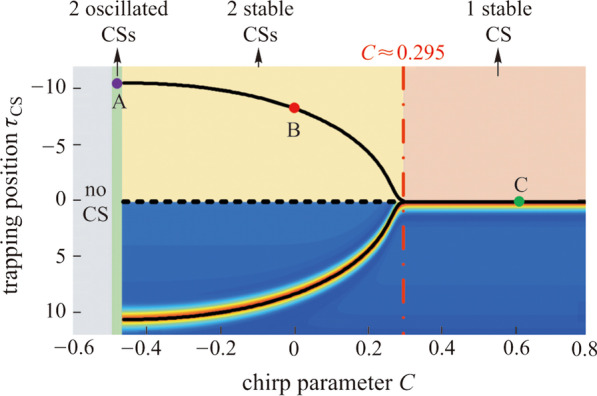

## Introduction

Continuous wave (CW) driven Kerr resonator has attracted considerable attention for its sustained dissipative Kerr cavity solitons (CSs) [1–4]. Relying on the double balance of Kerr nonlinearity and anomalous dispersion, as well as periodic pump and total losses, CSs manifest themselves as bright pulses with a sech-shape profile inside the cavity [1]. First observed in the macroscopic fiber ring resonator [4], CSs have been vigorously developed in the monolithic high-Q microresonator due to its compact volume, low power consumption, and complementary metal oxide semiconductor (CMOS) compatibility. Additionally, microresonator based optical frequency comb has wide applications in chip-scaled spectroscopy [5–7], optical clocks [8, 9], massively parallel coherent optical communication [10, 11], integrated frequency comb generator [12–14], astrocombs [15, 16], and fast detecting LIDAR [17, 18].

The CW pump laser coupled into the resonator will form a homogeneous background, where CSs can sit atop at any position. This characteristic is the reason why the number of CS is stochastic without any active control [19–23] or passive modulation [24–26]. Although CW driving requires no complex pump shaping, the small temporal overlap between the CS and the background leads to the low power conversion efficiency from pump to soliton [27]. To increase the conversion efficiency, some methods such as dark pulse generation [28], mutually coupled optical cavities [29], and the synchronized pulse pumping [27], have been proposed. The dispersion engineering and complicated dual-ring system are essential for the former two methods, respectively. For a present single cavity, synchronized pulse pumping can be the most effective method to enhance the conversion. Comparing to the CW condition, pump pulse will generate a pulse-shape background, which is an inhomogeneous amplitude base [30]. The trapping point where CSs tend to locate at is determined by such inhomogeneity, which has been experimentally demonstrated to generate deterministic single soliton or double soliton [27]. The corresponding physical mechanism called spontaneous symmetry braking (SSB), has been theoretically established in Ref. [30]. Distinct to the phase inhomogeneity that causes CS to always drift to the phase maximum [31], amplitude inhomogeneity attracts CSs to trap at specific positions related to the driving filed amplitude. Two trapping points under the condition of large driving pulse peak power will decrease the probability of single soliton generation, which is not a benefit to obtaining smooth spectral envelope. Furthermore, a velocity mismatch between CS and pump pulse also has significant impact on the bi-stability dynamics of Kerr resonators [32]. Therefore, a new method remains to be proposed to recover the SSB and reduce the impact of desynchronization.

In this paper, we report on the numerical investigations of CS dynamics under the pump condition of chirped optical pulses. The chirped pulses will simultaneously induce a phase and amplitude modulation on the background. By inducing a positive chirp parameter, the trapping positions of CS are closer to the pump pulse maximum, and CS can be robustly trapped at the peak with enough chirp value. Furthermore, the chirped pulses can enlarge the existence range of CSs driven by desynchronized chirped pulses. Our work not only gives new insights into the dynamics of CSs driven by chirped optical pulses, but also provides a feasible method to regulate the CSs in the passive Kerr resonators.

## Theoretical model

We consider injecting a series of chirped optical pulses into the anomalous dispersive Kerr resonator, whose second order dispersion is dominant corresponding to these experimental resonators [27, 33]. We focus on both synchronization and desynchronization, and the group velocity mismatch between the chirped pulses and CS is taken into account. The evolution of the slowly varying intracavity electric field envelope *E*(*z*,*τ*) is described by the dimensionless Lugiato–Lefever equation (LLE) [32, 34]:

1 $$\frac{\partial E(z,\tau )}{{\partial z}} = \left[ { - 1 + {\text{i}}(\left| E \right|^{2} - \Delta ) - {\text{d}}\frac{\partial }{\partial \tau } + {\text{i}}\frac{{\partial^{2} }}{{\partial \tau^{2} }}} \right]E + S(\tau ),$$

where *z* is the normalized propagation distance in the cavity. The *τ* is the fast time variable which describes the temporal profile over one roundtrip. The terms on the right-hand side of Eq. (1) are cavity losses, self-phase modulation, cavity phase detuning, group velocity mismatch, group velocity dispersion, and fast time-dependent driving field. The corresponding normalization is as follows: *z* = *αz'*, *τ* = *τ'*(2*α*/|*β*_2_|)^1/2^, *E* = *E'*(γ/α)^1/2^, Δ = *δ*_1_/α, *d* = Δ*β*_1_(|*β*_2_|/(2*α*^3^))^1/2^, *S*(*τ*) = *E*_in_(*τ*)(*γθ*/(*α*^3^*L*^2^))^1/2^. Here, *α* is the cavity losses per unit length, *β*_2_ is the group-velocity dispersion coefficient, *γ* is the self-phase modulation coefficient, *δ*_1_ is phase detuning per unit length, Δ*β*_1_ is the group velocity mismatch in the case of desynchronization, *E*_in_(*τ*) is the amplitude of the chirped pulses, *θ* is power coupling coefficient, and *L* is the total length of the Kerr resonator. In this work, we consider a chirped Gaussian pulse profile, *S*(*τ*) = *S*_0_exp[–(1 + j*C*) *τ*^2^/(2*τ*_g_^2^)], where *S*_0_, *C*, and *τ*_g_ represent the amplitude, chirp parameter and pulse width, respectively. We assume the pulse width *τ*_g_ is much larger than that of the CS ensuring a quasi-homogeneous background. Thus, the gradient induced by the chirped pulses can be regarded as perturbed terms acted on the CS.

To analyze the effects of the chirped optical pulses, we write *S*(*τ*) = *S*_0_exp(− *τ*^2^/(2*τ*_g_^2^)exp[j*ϕ*(*τ*)], *ϕ*(*τ*) =  − *Cτ*^2^/(2*τ*_g_^2^). Substituting the ansatz *E* = *E'*exp[j*ϕ*(*τ*)], Eq. (1) can be written as

2 $$\begin{gathered} \frac{\partial E(z,\tau )}{{\partial z}} = [ - \alpha_{{\text{R}}} + {\text{i}}(\left| E \right|^{2} - \Delta_{{\text{R}}} ) - (2\phi^{\prime} + {\text{d}})\frac{\partial }{\partial \tau } + {\text{i}}\frac{{\partial^{2} }}{{\partial \tau^{2} }}]E \\ + S_{0} \exp \big(\frac{{ - \tau^{2} }}{{2\tau_{{\text{g}}}^{2} }}\big), \\ \end{gathered}$$

where *ϕ′* = d*ϕ*(*τ*)/d*τ*, *ϕ″* = d^2^*ϕ*(*τ*)/d*τ*^2^, *α*_R_ = 1 + *ϕ″*, and Δ_R_ = Δ + *ϕ′*^2^ + d*ϕ′*. The total drift velocity *v* of the CS relative to the chirped pulse can be described as

3 $$v = \left. {a[\text{Re}(S),\Delta_{{\text{R}}} ]\frac{{{\text{d}}[\text{Re}(S)]}}{{{\text{d}}\tau }}} \right|_{{\tau = \tau_{{\text{g}}} }} + 2\phi^{\prime} + d.$$

The proportionality coefficient *a* is the projection of the CS’s neutral mode along a linear fast time variation, which is determined by Re(*S*) and Δ_R_ [30]. In this way, we could regard the chirped driving field as the coexistence of the phase and amplitude pulses. Since the value of chirp para-meter *C* we consider is small (*C* ≤ 1), the CS here is without distortion due to *ϕ″* ≤ 0.02 ≪ 1 and *ϕ′*^2^ + d*ϕ′* ≪ Δ.

## Results and discussion

To investigate the CS dynamics, we integrate the Eq. (1) with the split-step Fourier method, whose step is set to be the normalized cavity length. We set the amplitude *S*_0_ = 2.3 > 1.98, which is an amplitude threshold of SSB [30]. The chirp parameter *C* and the pulse width *τ*_g_ are fixed to be 0.6 and 15, respectively. We linearly scan the phase detuning Δ with a step of 0.001 till Δ = 4.

**Fig. 1:**
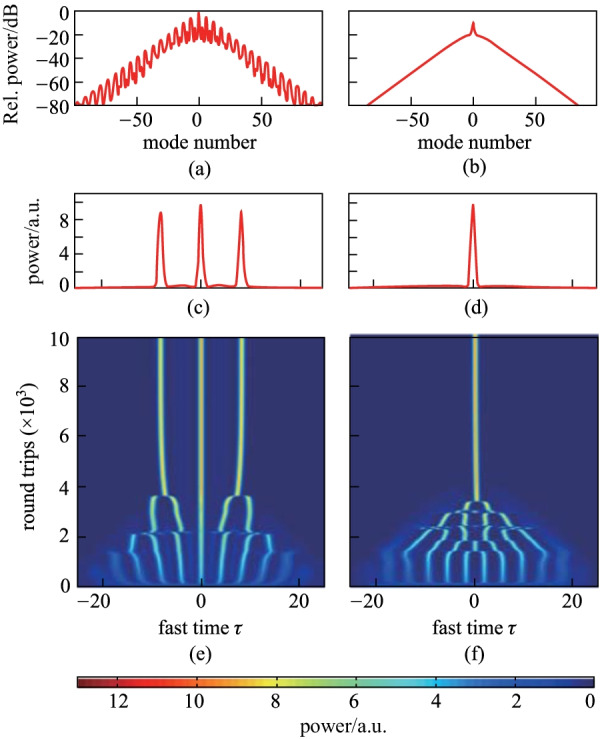
**a** and **b** Spectral profiles at a stable CS state (10^4^ round trips) generated by the amplitude pulses and chirped pulses, respectively. **c** and **d** Temporal profiles corresponding to **a** and **b**, respectively. **e** and **f** Evolutions of the intracavity field with amplitude pulse and chirped pulse driving fields, respectively

Figure 1 shows the comparison of stable CS state generated by  amplitude pulse and chirped pulse driving fields. The evolutions of the intracavity field shown in Fig. 1e and f contain the particular nonlinear states including Turing pattern, chaotic waves and soliton. We can find that triple CSs  can stably survive after collisions as shown in Fig. 1e. This is consistent with the analysis in Ref. [30], indicating there are another two trapping points besides the peak point. Figure 1f shows a distinct result that only a single CS sits atop the peak point of the background indicating one trapping position. The spectrum shown in Fig. 1a has envelope modulations due to the interference between the CSs shown in Fig. 1c. The single soliton shows a rather smooth spectrum as is illustrated in Fig. 1b and d. It can be explained as follows. At the trailing edge of the chirped pulse, the CSs is temporally advanced for going through a positive drift velocity *v* (*v* = 2*ϕ′* + *a*·d[Re(*S*)]/d*τ* > 0); at the leading edge, the CSs is temporally delayed for going through a negative drift velocity (*v* = 2*ϕ′* + *a*·d[Re(*S*)]/d*τ* < 0). In this way, CSs can be robustly trapped at the peak point of the chirped pulse.

**Fig. 2:**
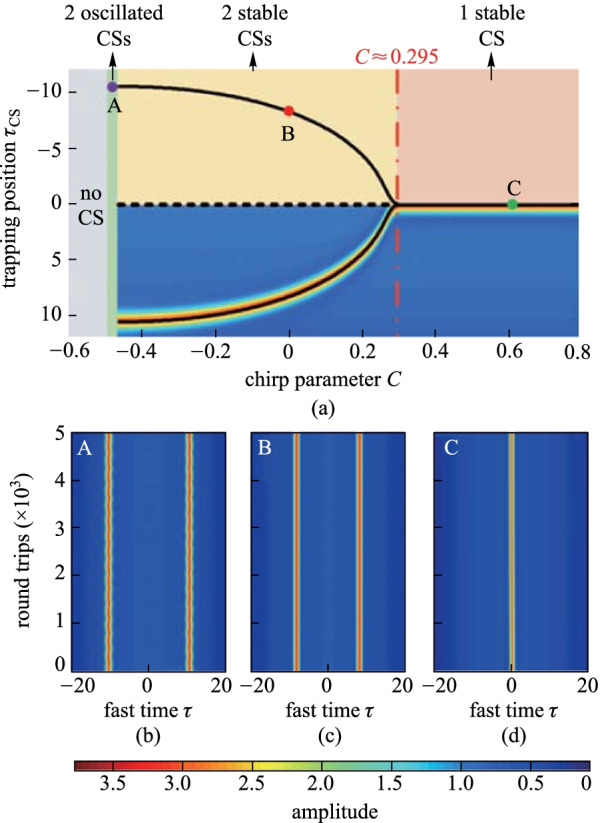
**a** Trapping position *τ*_CS_ as a function of the chirp parameter *C* under a chirped pulse driving condition with *S*_0_ = 2.3, *τ*_g_ = 15, and Δ = 4. Black curves show the steady-state positions of the CS for asymmetric (two CSs) and symmetric (single CS) states. The black dashed line indicates the unstable peak point. Red dash-dot line indicates the critical chirp parameter for single soliton generation. The regions with different colors from left to right correspond to no CS, two oscillated CSs, two stable CSs and a single stable CS, respectively. The corresponding dynamics is shown in the lower half of **a**. **b**−**d** Intracavity dynamics correspond to the points A, B, and C in **a**

To quantitively investigate the effects of the chirp, we run the simulations to figure out the continuous variation of the trapping positions along with the chirp parameter. We add an initial condition depicting an approximated soliton profile *E*(*z* = 0, *τ*) = (2Δ)^1/2^sech[Δ^1/2^(*τ* − *τ*_0_)] on the stable quasi-continuous background, where the initial temporal location *τ*_0_ is set to be 10. Figure 2a shows the trapping temporal positions *τ*_CS_ varies with the chirp parameter *C*. The diagram is divided into several areas with different colors from left to right, corresponds to no CS (gray), two oscillated CSs (green), two stable CSs (cream) and a single stable CS (pink), respectively. The blue region in Fig. 2a is the corresponding dynamics of the CSs, which is depicted by recording a complete stable soliton state per roundtrip. The trapping temporal position *τ*_CS_ without the chirp is observed to be 8.179, where the proportional coefficient *a* is zero. When the chirp parameter is negative, the trapping position drifts along the gradient (*ϕ*′ ) of the chirp pulse. Since the coefficient *a* changes its sign to be opposite to the chirp *C*, the CS can exist at specific position by satisfying the condition of 2*ϕ′* =  − *a*·d[Re(*S*)]/d*τ*. By increasing the chirp parameter *C*, the trapping position continuously drifts closer to the peak point till they coincide. We can observe the critical chirp value is 0.295, beyond which the CS can always locate at the peak point. We call this phenomenon as the recovery of SSB. In addition to the stable trapping position, the CSs exhibit temporal oscillations around the point where their existence would be expected to cease under conditions of CW driving, as illustrated in Fig. 2b, which has been demonstrated in the desynchronized amplitude pulse driving [32]. Therefore, with a suitable chirp parameter, deterministic single soliton can be obtained in a high amplitude driving condition.

**Fig. 3:**
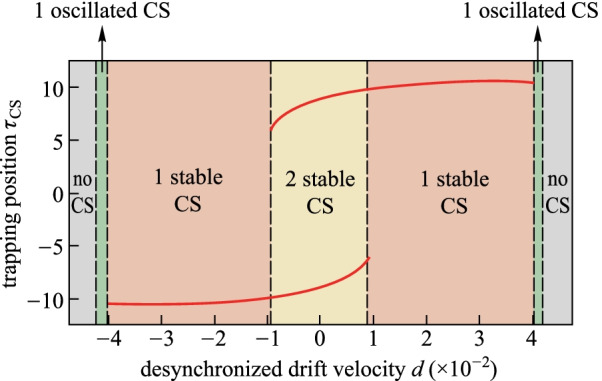
Trapping position *τ*_CS_ as a function of the desynchronized drift velocity *d* without chirp (*S*_0_ = 2.3, *τ*_g_ = 15, and Δ = 4), as is described by the red curves. The regions with different colors correspond to no CS (gray), one oscillated CS (green), one single stable CS (pink) and two stable CSs (cream), respectively

The above investigations concentrate on the synchronized pulse driving, and desynchronization will bring new dynamics and trapping positions.  To demonstrate that the stability and existence of the CSs are just determined by the modification of the background, we first study the impact of desynchronization without chirp. As is shown in Fig. 3, we can observe the same phenomena such as two stable CSs, a single CS and one oscillated CS, as that shown in Fig. 2, which indicates that the chirp and desynchronization only alter the trapping position *τ*_CS_.

**Fig. 4:**
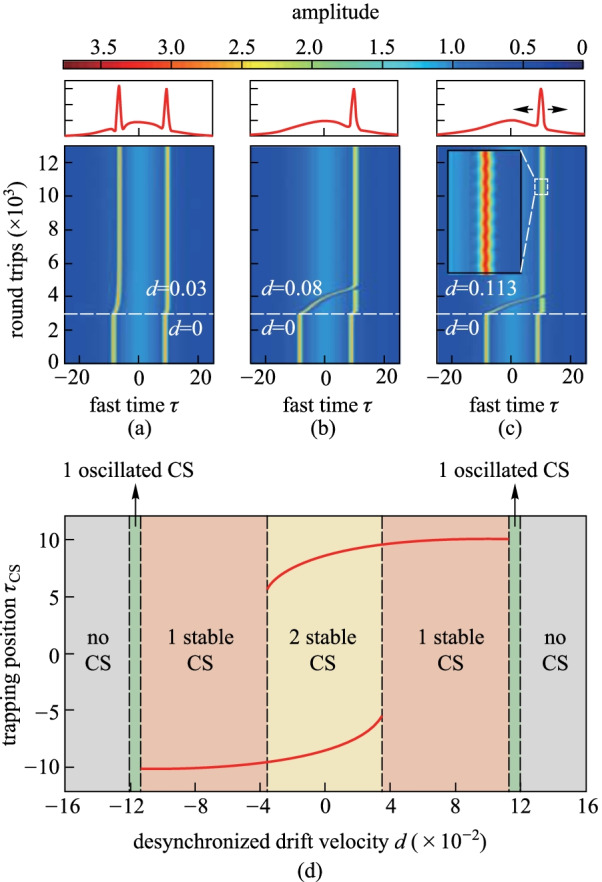
**a–c** Intracavity dynamics correspond to different desynchronized drift velocity. And distinct soliton states are finally obtained as is shown in the top panels. **d** Trapping position *τ*_CS_ as a function of the desynchronized drift velocity *d* under a chirped pulse driving condition of *S*_0_ = 3, *τ*_g_ = 10, and Δ = 4, as is described by the red curves. The regions with different colors correspond to no CS (gray), one oscillated CS (green), one single stable CS (pink) and two stable CSs (cream), respectively

To give a deeper insight into the impacts of the desynchronization, we change the physical parameters of the driving pulse as *S*_0_ = 3 and *τ*_g_ = 10. Figure 4a shows the simulation results, which illustrate the intracavity dynamics with a weak chirp parameter (*C* = 0.1). There is no initial desynchronization (*d* = 0) and two CSs are excited to locate at both sides of the background. At the 3000th round trip, a desynchronized drift (*d* = 0.03) is introduced and both CSs drift toward the leading edge of the chirped pulse till they arrive at a new trapping position. When increasing of the desynchronized drift to *d* = 0.08, as shown in Fig. 4b, the CS at the trailing edge (trailing CS) will first drift toward the leading edge and subsequently travel across the peak point. At the time the trailing CS gets closer to the CS at the leading edge, the leading CS ceases to exist due to the collision. At last, the trailing CS replaces the leading one and stays stable at the trapping position.

A more detailed analysis of the impact of desynchronization on the trapping position is shown in Fig. 4d. The solid red curves show the steady-state trapping positions of CS as a function of the desynchronized drift velocity *d*. Considering a relatively small drift velocity *d*, the trapping position still exhibits bi-stability, where two CSs can sit atop the background wave with asymmetric positions [cf. Figure 4a]. With a large drift velocity *d*, a single CS can survive on either trailing or leading edge of the background wave. Similar to the situation shown in Fig. 2b, the drifted CS also exhibits a temporal oscillation around a point (cf. Fig. 4c). Beyond the specific desynchronized drift velocity *d* =  ± 0.113, the CSs cease to exist as they are pushed below the minimum sustained driving amplitude.

**Fig. 5:**
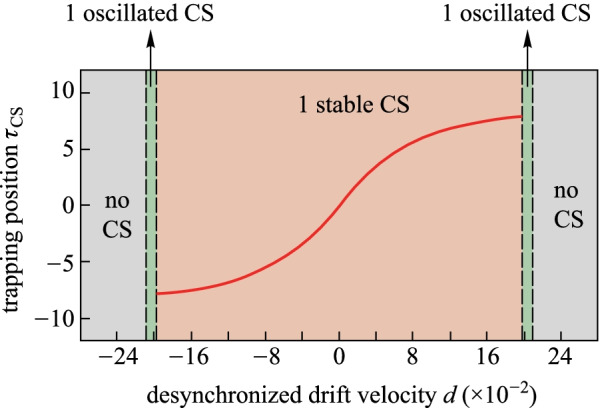
Trapping position *τ*_CS_ as a function of the desynchronized drift velocity *d* under a chirped pulse driving condition of *S*_0_ = 3, and *τ*_g_ = 10, as is described by the red curves. The regions with different colors correspond to no CS (gray), one oscillated CS (green), and one single stable CS (pink), respectively

The results in the case of large chirp parameter are distinct to that in Fig. 4. We increase the chirp parameter *C* to be 1, which is beyond the critical chirp value to ensure the CS can be trapped at the peak point of the background wave. Figure 5 shows the trapping position as a function of desynchronized drift velocity for *C* = 1. Distinct to the results shown in Fig. 4, the bi-stability region of the trapping position does not exist, which indicates the one-to-one correspondence between the trapping position and the desynchronized drift velocity. The deterministic single soliton state can also be obtained even in the desynchronization. In addition, comparing the existence range of CSs shown in Figs. 4 and 5, we can find that the pulse driven with larger chirp parameter has a wider existence range, which indicates that chirp is an effective way to reduce the desynchronized impacts on the CS survival.

## Conclusion

In conclusion, we have performed systematically theoretical and numerical investigations on the dynamics of CS driven by synchronized and desynchronized chirped optical pulses. To eliminate the multiple temporal trapping positions induced by the SSB under synchronized pulse driving, we introduced sufficient chirp to obtain one single trapping position for deterministic single soliton generation. In the case of desynchronization, our results show that no chirp and a weak chirp divide the trapping position diagram into several areas with different soliton states, where multiple (two) soliton states still exist. We demonstrate that a sufficient chirp value can help obtain the deterministic single soliton state and enlarge the CS existence range under the desynchronized pulse driving.

**Fig. 6:**
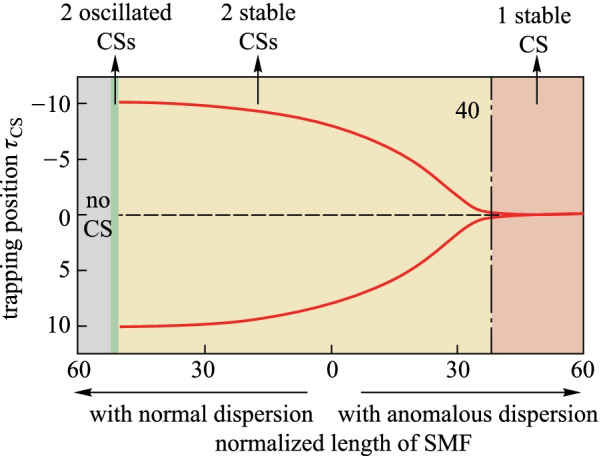
Trapping position *τ*_CS_ as a function of the normalized length of SMF under a chirped pulse driving condition of *S*_0_ = 2.3, *τ*_g_ = 15, and Δ = 4. Red curves show the steady-state positions of the CS for asymmetric (two CSs) and symmetric (single CS) states. The horizontal ordinate represents the normalized length of SMF, the region designated by right and left arrows represent the SMF with anomalous dispersion and equally normal dispersion, respectively

Experimentally, to obtain the chirped optical pulses, we consider the situations for the micro- and macroscopic cavity. For the macroscopic cavity (~ MHz repetition rate), the optical pulses with 1 ns pulse width can be generated by the the amplitude modulator driven by a 1 GHz pattern generator, which can effectively avoid the build-up stimulated Brillouin scattering [33]. For the microcavity, the optical pulses with a short width and high repetition rate can be obtained according to the reported method described as: the continuous wave laser first passes through the intensity modulation to carve out the modulation half-period with correct chirp sign. It then is strongly chirped by using a phase modulator driven by a 20 GHz scale microwave source and compressed into picosecond pulses via linear propagation in a chirped fiber-Bragg grating [27]. Optical pulses with higher repetition rate (> 100 GHz) can be generated in the semiconductor laser [35]. After generating the optical pulses, the chirp can be induced by injecting the optical pulses into a segment of single mode fiber (SMF). Illustrating the trapping position varying with the length of SMF with anomalous dispersion, Fig. 6 shows the trapping position *τ*_CS_ as a function of the normalized length of zero-loss SMF with anomalous dispersion and equally normal dispersion. As can be seen, the trapping position continuously drifts closer to the zero point with increasing the length of SMF with anomalous dispersion. This estimation is consistent with the numerical results shown in Fig. 2. The critical length of SMF (critical chirp value) is 40 corresponding to realistic 52.8 km SMF (10 dB loss), which refers to the resonator with a cavity length of 100 m and a finesse of 21.

Our findings reveal the nonlinear dynamics of CS driven by chirped optical pulses in the Kerr resonator, which can be complementary research on the CS property in the inhomogeneous driving field. Moreover, the method we proposed can be effective to reduce the impacts of desynchronization and obtain a deterministic single soliton driven by higher amplitude pulses.
